# Low-Volume Toolbox for the Discovery of Immunosuppressive Fungal Secondary Metabolites

**DOI:** 10.1371/journal.ppat.1003289

**Published:** 2013-04-11

**Authors:** Erwin Berthier, Fang Yun Lim, Qing Deng, Chun-Jun Guo, Dimitrios P. Kontoyiannis, Clay C. C. Wang, Julie Rindy, David J. Beebe, Anna Huttenlocher, Nancy P. Keller

**Affiliations:** 1 Department of Medical Microbiology and Immunology, University of Wisconsin-Madison, Madison, Wisconsin, United States of America; 2 Department of Biomedical Engineering, University of Wisconsin-Madison, Madison, Wisconsin, United States of America; 3 Department of Pharmaceutical Science, University of Southern California, Los Angeles, California, United States of America; 4 The University of Texas MD Anderson Cancer Center, Houston, Texas, United States of America; University of Massachusetts Medical School, United States of America

## Abstract

The secondary metabolome provides pathogenic fungi with a plethoric and versatile panel of molecules that can be deployed during host ingress. While powerful genetic and analytical chemistry methods have been developed to identify fungal secondary metabolites (SMs), discovering the biological activity of SMs remains an elusive yet critical task. Here, we describe a process for identifying the immunosuppressive properties of *Aspergillus* SMs developed by coupling a cost-effective microfluidic neutrophil chemotaxis assay with an *in vivo* zebrafish assay. The microfluidic platform allows the identification of metabolites inhibiting neutrophil recruitment with as little as several nano-grams of compound in microliters of fluid. The zebrafish assay demonstrates a simple and accessible approach for performing in vivo studies without requiring any manipulation of the fish. Using this methodology we identify the immunosuppressive properties of a fungal SM, endocrocin. We find that endocrocin is localized in *Aspergillus fumigatus* spores and its biosynthesis is temperature-dependent. Finally, using the *Drosophila* toll deficient model, we find that deletion of *encA*, encoding the polyketide synthase required for endocrocin production, yields a less pathogenic strain of *A. fumigatus* when spores are harvested from endocrocin permissive but not when harvested from endocrocin restrictive conditions. The tools developed here will open new “function-omic” avenues downstream of the metabolomics, identification, and purification phases.

## Author Summary

Several fungal pathogens produce bioactive small molecules, commonly known as secondary metabolites (SMs) that contribute towards disease development in susceptible hosts. Genome assessment of human pathogenic *Aspergillus* species indicates these fungi have the capabilities of producing hundreds of SMs, most of which are currently not characterized for their effect on human health and the immune system. This lack of knowledge is directly correlated to the difficulties of obtaining assayable quantities of pure metabolites. To overcome this roadblock in assessing the potential impact of SMs on the immune system, our laboratories have developed a two-tiered cost-effective, high-throughput program utilizing microfluidic platforms and a novel zebrafish model to identify SMs inhibiting neutrophil chemotaxis. Using minimal and physiologically relevant amounts of SMs, this systematic approach has identified the *A. fumigatus* spore SM, endocrocin, as a potent chemotaxis inhibitor. Interestingly, the production of endocrocin is temperature dependent and virulence studies with the endocrocin null mutant implicates the temperature at which the fungus forms spores as a factor in disease development.

## Introduction

The secondary metabolome provides filamentous fungi with a biologically active panel of molecules, deployed in the presence of competing/host organisms or specific microenvironmental factors, and increasingly found to afford both physical and competitive fitness to the producing fungus [1]. Although the study of fungal secondary metabolism has reached the ‘omics era, with the development of tools for efficient genetic exploration [2] and the improvement of HPLC and LC-MS methods [3], a significant challenge remains in identifying the biological activity of the purified compounds. The minute quantity of metabolites (nano- to micrograms) collected from the latter methods and the large number of isolated compounds (hundreds to thousands) are important limiting factors in this endeavor. Thus, as the methods for identifying SM gene clusters and the compounds they produce are becoming well established [4], there is an increasing need for improved assays, compatible with the fungal metabolomics process, that can reveal the biological activity of metabolites produced and break a bottleneck in scientific advancement.

*Aspergillus spp*. SMs are of particular interest in medical research as the genus is genetically accessible, produces a plethora of bioactive compounds [5], and contains several opportunistic pathogenic species including *A. fumigatus* and *A. nidulans* whose SMs are assessed in this study [6], [7]. Though it is likely that a number of factors together contribute in making these species effective pathogens, SMs play an important role in the virulence of *Aspergillus*-related diseases as direct toxins and modulators of the immune response [8], [9]. As the innate immune response is the primary line of defense against fungal spores in the lung, inhibition of essential functions of these cells may confer to the fungi an ability to evade immune clearance, and increase its pathogenicity. These findings highlight the necessity of mapping the interactome between fungi and host organisms to establish the pathomechanism of fungal diseases as well as to bioprospect.

Leading to this current study are the series of works showing LaeA, a global regulator of secondary metabolism to be a virulence factor not only in pathogenic Aspergilli [10]–[13] but in all filamentous pathogenic fungi assessed to date [12], [14], [15]. Examination of the *laeA* mutant in *A. fumigatus* implicated unidentified SMs in development of invasive aspergillosis [10], [11]. As several studies have shown *A. fumigatus* culture filtrates to inhibit neutrophil chemotaxis [16]–[18], we considered it possible that LaeA-regulated SMs could be chemotaxis inhibitors. Complicating this hypothesis, however, is the fact that LaeA regulates dozens of SM clusters, all of which can produce multiple derivatives from the same biosynthetic pathway, whose purification results in small available quantities [19].

Traditional *in vitro* neutrophil migration models, often performed in well-plates, do not allow a good level of control over the migration microenvironment, do not allow imaging of the cells during the migration process, and require large amounts of purified compound (micrograms to grams in hundreds of microliters to milliliters). Advances in microscaled assays have demonstrated enabling characteristics, with the ability to use microliters or less of reagents, develop high-throughput applications, and design assays with more control over the micro-environment [20]. The development of open systems, that interface with existing fluid handling equipment, contributed in making microscaled assays more accessible and better suited for screening libraries of individual compounds [21]. Further, these approaches enable the development of functional cell-based assays, such as arrayed leukocyte recruitment assays [22]. However, these have not been applied for identifying SMs modulating the fungal-immune interaction, nor for screening of fungal SMs.

Appropriate *in vivo* models are also required to support *in vitro* progress. Current models such as *Galleria* [23] or *Drosophila* [24], are accessible and inexpensive but do not fully recapitulate the vertebrate immune system. More relevant models such as the murine model are logistically challenging, require excessive amounts of purified SMs, and are still fraught with deficiencies in visualization of innate cell response [25]. Recently, the development of the zebrafish embryo model has proven to be a vertebrate model well suited for leukocyte studies as it is readily accessible, small, and transparent and has been established as a model system for infectious diseases including fungi [26].

Here, we demonstrate a two-tiered screening approach for identifying the immunosuppressive and neutrophil recruitment inhibitory activity of LaeA-regulated SMs, capitalizing on advances of microscale *in vitro* systems and an original zebrafish model. An arrayed microfluidic *in vitro* neutrophil recruitment platform was developed, compatible with manual and automated pipettes, allowing for rapid assessment of the neutrophil recruitment inhibition properties of purified *Aspergillus* SMs. Passive open microfluidic methods were employed for creating arrayed gradient-generation devices operable in typical biological laboratory settings and minimizing reagent use. Bioactive metabolites identified from this platform were then assessed in an *in vivo* zebrafish recruitment assay. The zebrafish assay is enabling as it significantly reduces the quantity of compounds required and provides a quickly assessed window into innate immune response. Using this approach, we report the identification of the neutrophil recruitment inhibition activity of endocrocin, an *A. fumigatus* SM, and further characterized its localization in spores of the growing fungus.

## Results

### Microfluidic gradient-generation device

**Figure 1:**
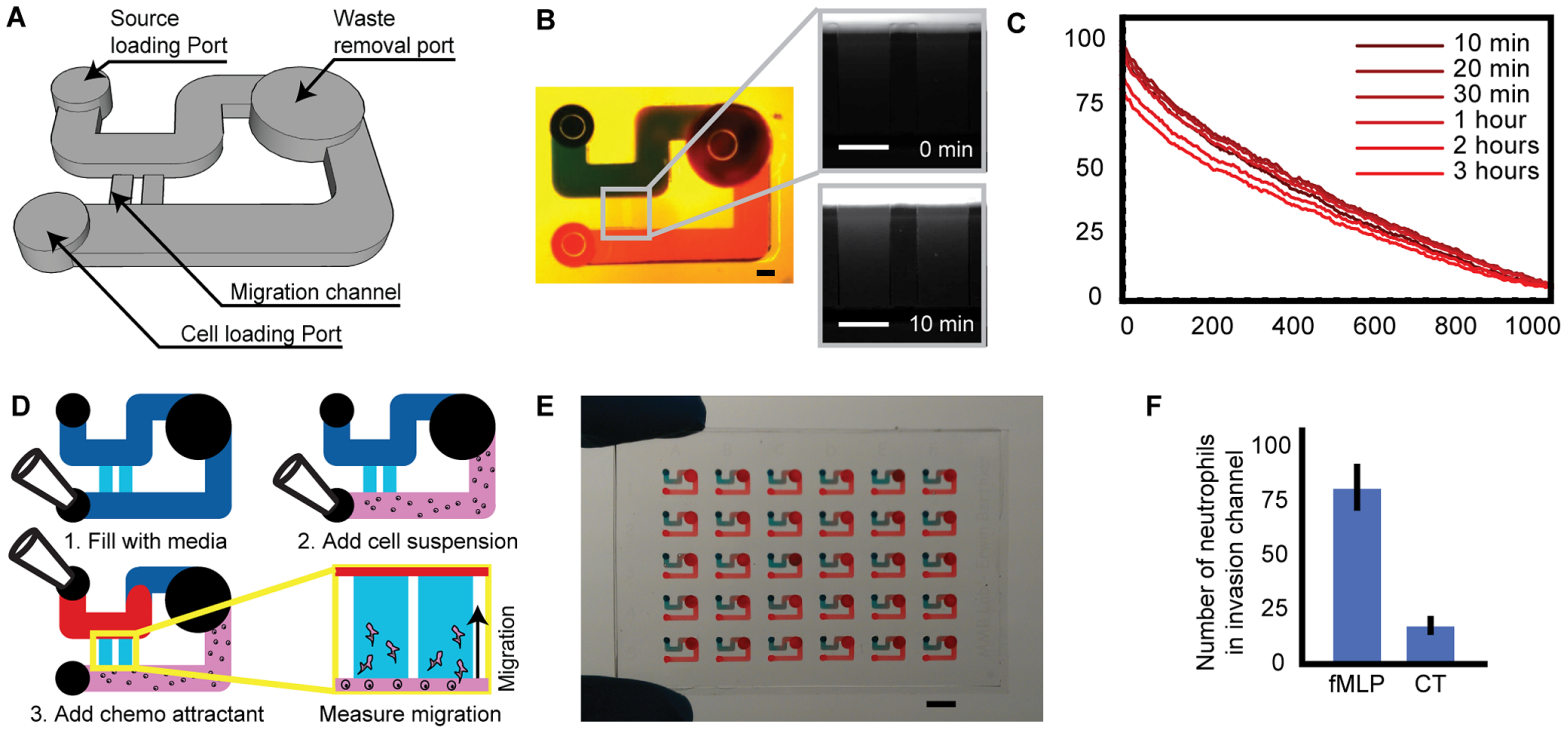
Microfluidic device for static gradient generation and neutrophil migration assessment. (A) Schematic of an individual microfluidic gradient generation and neutrophil migration device. Neutrophils placed in the sink reservoir of the channel can sense a gradient developing in the migration channel and displace towards and into that channel (B) Illustration of the device filled with red food colorant and blue food colorant placed in the chemoattractant source channel. Inserts are fluorescent microscopy images of Alexa488 diffusing into the channels. Scale bar = 300 µm (C) Quantification of the gradient formation in the migration channels. (D) The device can be loaded simply in 3 steps with a pipette. (E) Illustration of a 6 by 5 array of devices. Scale bar = 3 mm (F) Quantification of the number of neutrophils that have invaded the migration channel in response to a known chemoattractant, fMLP, compared to a negative control.

In order to systematically test the large number of compounds provided by liquid chromatography methods, a microscale platform for assessing neutrophil chemotaxis properties was designed. The use of tubeless microfluidic-interfacing methods minimizes dead volumes and static gradient-generation methods further minimizes volume requirements [22], [27]. Thus, neutrophil recruitment can be assessed with as little as 3 µL of purified compound, using only a simple micropipette. In this approach, the gradient is generated in a reproducible way by leveraging a flow bypassing method based on ensuring that no undesired flow passes through the gradient channel, rather a second flow path of significantly lesser fluidic resistance diverts the bulk of the flow (Figure 1A,B). The reliability of the fluidic handling mechanism used, and the simplicity of the design, enables rapid loading protocols and large arrays of microdevices. In order to achieve the highest throughput possible, batch-processing algorithms were developed, enabling the quantification of neutrophil migration properties from an endpoint phase-contrast image of the migrating neutrophils. Together, these advances allow the processing by hand of up to 300 migration data-points per experimental run in an embodiment that can be readily interfaced using handheld pipettes and automated liquid handlers.

The static gradient-generation device was verified using fluorescent dyes and food colorant (Figure 1B), demonstrating that the gradient establishes in minutes as assessed by fluorescent imaging of AlexaFluor488 (Figure 1C). The microfluidic gradient, once established, was able to maintain steady for several hours, allowing the reliable measure of neutrophil (or other leukocyte) migration over the course of a typical neutrophil experiment (Figure 1C). The microfluidic channel can be entirely prepared in 3 simple pipetting steps, which require seconds, and can be interface-able with electronic and robotic liquid handlers (Figure 1D). Using the flow generation method described, as well as the static gradient-generation methods, we demonstrate the ability to create large arrays of gradient devices which can be prepared in large batches allowing the operation of several hundred per day (Figure 1E). As these contain very little dead volumes (being devoid of tubes and actuation equipment) a single blood draw of 10–20 mL is sufficient to operate thousands of these channels, provided the appropriate liquid handling and data acquisition equipment is used.

### In vitro neutrophil recruitment inhibition assay

**Figure 2:**
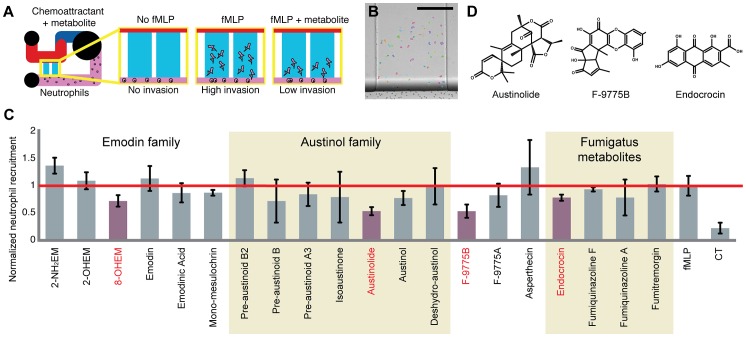
Fungal SM recruitment inhibition screen. (A) Schematic of the screening principle to identify secondary metabolites inducing neutrophil migration inhibition. (B) Example of microscope image resulting from a migration experiment with the outlines produced by the automated counting algorithm. Scale bar = 300 µm (C) Screening of *A. nidulans* and *A. fumigatus* purified secondary metabolites for human primary neutrophil migration inhibition properties. Displayed are the number of recruited neutrophils to a mix of fMLP and 10 µM concentration of compound normalized to the positive fMLP control. Compounds identified in red significantly reduce migration towards fMLP (p<0.05). 2-NH_2_EM = 2 amine emodin, 2-OHEM = 2 hydroxyl emodin, 8-OHEM = 8 hydroxyl emodin, CT = control. (D) Compounds selected for *in vivo* screening.

In order to systematically test the large number of compounds provided by liquid chromatography methods, the microscale platform was used to screen for compounds inhibiting neutrophil recruitment. The purified compounds were introduced in conjuncture with a known chemoattractant (fMLP) in the gradient-source reservoir, causing the recruitment of neutrophils into the gradient microchannels (Figure 2A,B). A decrease in the number of neutrophils recruited compared to the positive control suggests an inhibitory effect and is considered as a positive hit for the screen. Using this method, a set of 20 purified compounds (each inserted in a volume of 3 µL at 10 µM, n = 9) from *A. nidulans* and *A. fumigatus* was screened (Figure 2C). Results show that four compounds – 8-hydroxyl emodin, austinolide, F-9775B, and endocrocin – display a significant decrease in neutrophil migration.

### Zebrafish neutrophil recruitment inhibition assay

**Figure 3:**
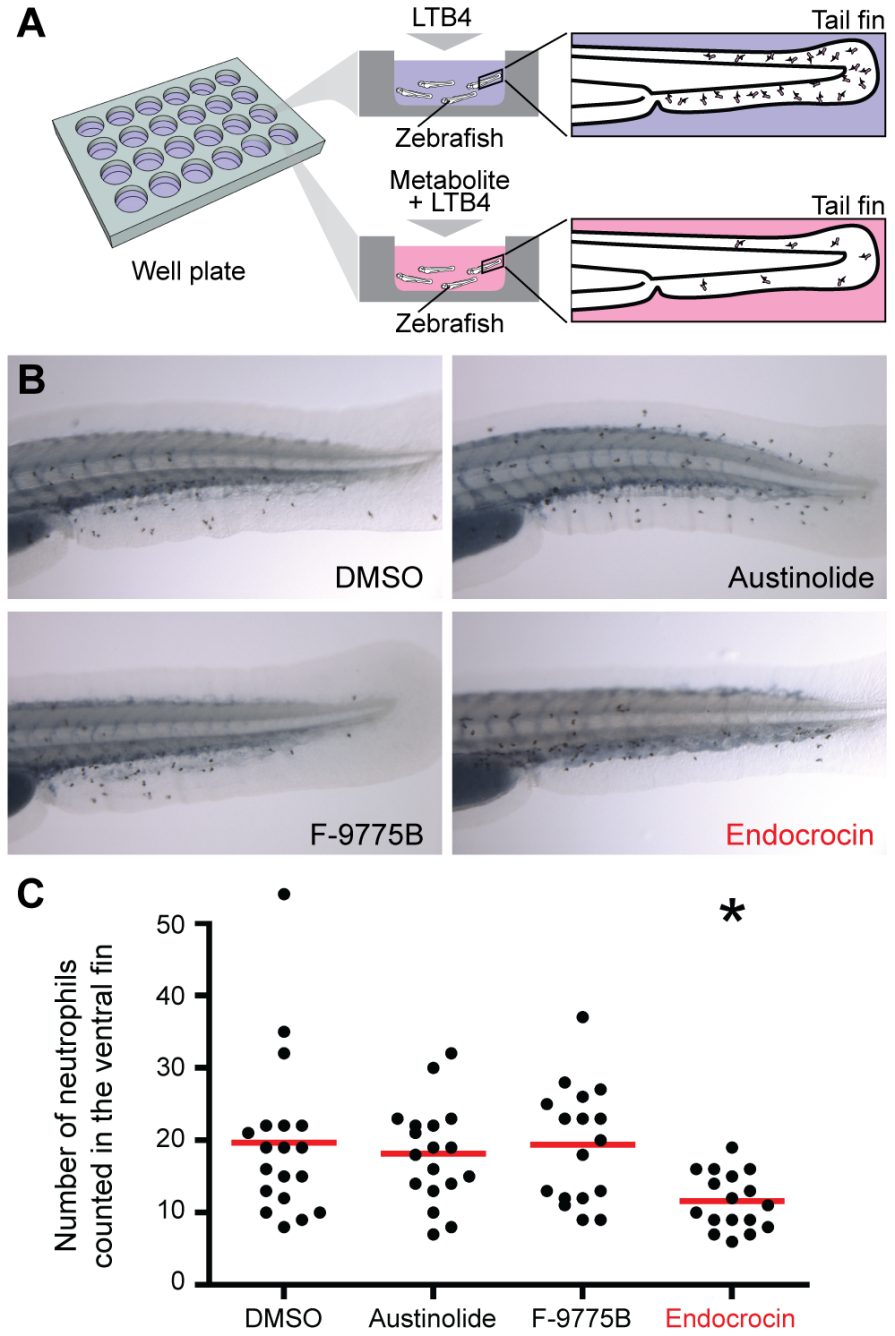
In vivo screening of fungal SMs in a wound-free zebrafish screening model. (A) Schematic of the zebrafish neutrophil recruitment inhibition assay. (B) Stereomicroscope images of fixed and stained fish showing neutrophil recruitment in the tail fin for 3 compounds each applied at a concentration of 10 µM, compared to the negative control (DMSO). (C) Quantification of the neutrophils recruited in the tail fin of zebrafish (*p-value<0.05, the graph is a representative graph of the 3 repeats performed).

As *in vitro* assays do not account for the complexity of whole organisms, we developed a low-volume *in vivo* neutrophil recruitment assay based on advances in zebrafish models. Zebrafish are a very attractive model for developing neutrophil research assays as they are transparent, allow the observation of immune cells *in vivo*, low-cost, and importantly have a strong immunological resemblance to the human system [26]. However, most neutrophil recruitment assays in zebrafish to date were performed through a wounding assay in which the fish is wounded by a needle stab or a cut in the fin or the body [28]. This approach has been successful but requires the individual manipulation of each fish, which is not amenable to screening applications. We developed a neutrophil recruitment inhibition assay based on the ability of a known chemoattractant, leukotriene B4 (LTB_4_), to diffuse through the skin of the zebrafish and induce neutrophil recruitment out of the Caudal Hematopoietic Tissue (CHT). In conjuncture with a neutrophil migration inhibitor added to the well in which the zebrafish are bathing, the level of recruitment can be reduced and easily assessed (Figure 3A). We validated this assay using LY294002, a known neutrophil chemotaxis inhibitor targeting PI3K, and found that neutrophil recruitment to the CHT was entirely suppressed (Movie S1 and S2).

This assay is rapid and accessible as many zebrafish are treated simultaneously in a multi-well plate and it does not require the use of wounding or micro-injection methods. Furthermore, the fish can be readily fixed and neutrophils quantified using simple optical microscopy (Figure 3B). Based on availability, three of the four compounds identified in the *in vitro* screen were tested for their inhibitory properties to neutrophil chemotaxis. Results show that only endocrocin displayed a significant reduction in the number of neutrophils recruited to the tail fin (Figure 3B,C). We further assessed the general cytotoxicity of the compound, and did not observe a reduction in zebrafish survival after 4 days at endocrocin concentrations up to 10 µM (data not shown). Similarly, endocrocin did not induce an increase in neutrophil death in the timescale and concentrations used in the microfluidic assays (Figure S1).

### Endocrocin is a temperature-dependent conidial metabolite

**Figure 4:**
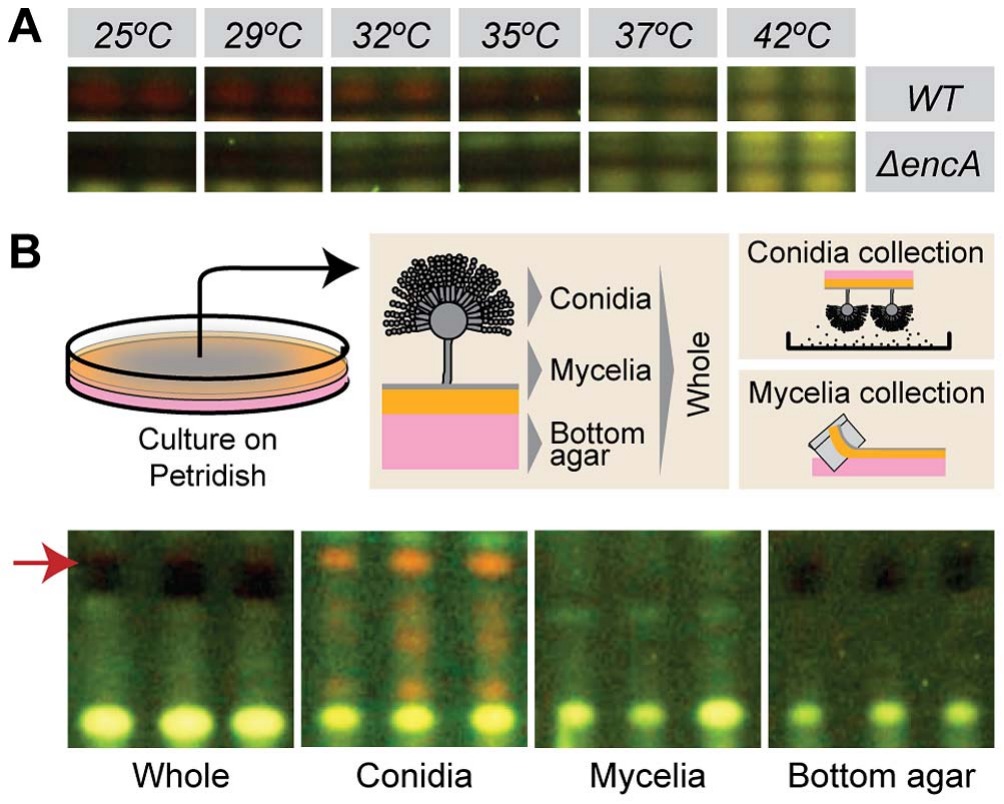
Endocrocin is a spore metabolite whose production is temperature dependent. (A) Thin layer chromatography (TLC) of crude extracts from *A. fumigatus* WT and *ΔencA* at 25, 29, 32, 35, 37, and 42°C. (B) Tissue-specific extraction of conidia, mycelia, and bottom agar of *A. fumigatus* WT, and TLC analysis of the metabolite profile. The red arrow represents the endocrocin standard.

We investigated properties of endocrocin production in order to gain insight on its mode of interaction with a host. Endocrocin is a LaeA regulated anthraquinone whose biosynthesis pathway was recently identified in *A. fumigatus* [29]. Given the potential role of endocrocin for modulating the immune response, we sought to characterize the mode of production and tissue specificity of this SM. We analyzed the production of endocrocin in both the WT strain and a *ΔencA* strain of *A. fumigatus* grown on GMM-agar at temperatures varying from 25°C to 42°C (Figure 4A). At temperatures below 35°C, the WT fungus produces endocrocin, while at higher temperatures it does not. As expected, the *ΔencA* strain did not produce endocrocin. The location of endocrocin production was characterized by analyzing crude extracts from different fractions of the fungal culture grown on solid medium: the conidia, the mycelia (top agar), and secreted metabolites (bottom agar). Endocrocin was only observed in the conidial fractions and not in the fractions containing mycelia (top agar) or soluble secreted factors (bottom agar, Figure 4B).

### Functional range of endocrocin-induced neutrophil inhibition

**Figure 5:**
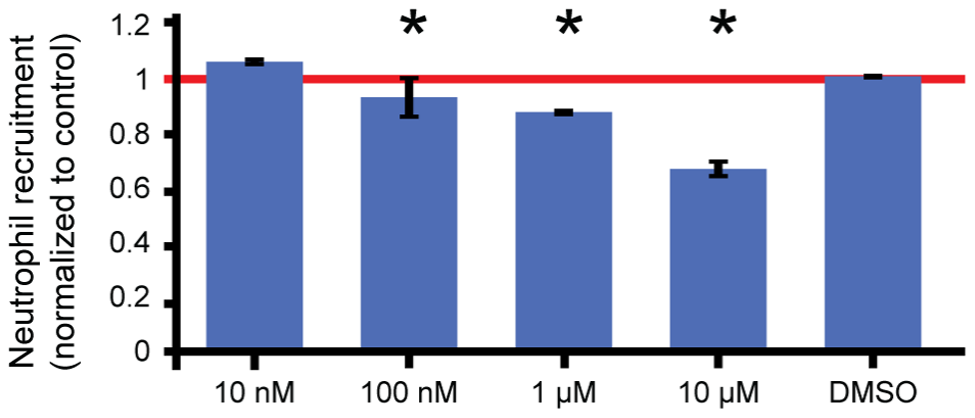
Endocrocin dose-response. Dose response of human primary neutrophil recruitment inhibition in the microfluidic platform for endocrocin (*p-value<0.05).

We identified the functional range of endocrocin inhibition by performing a dose response assay of endocrocin using the microfluidic neutrophil migration platform. Results show that a significant reduction of neutrophil inhibition can be observed at concentrations as low as 100 nM, with an inhibition of up to 40% for concentrations of 10 µM (Figure 5).

### Virulence in the Toll-deficient Drosophila model

**Figure 6:**
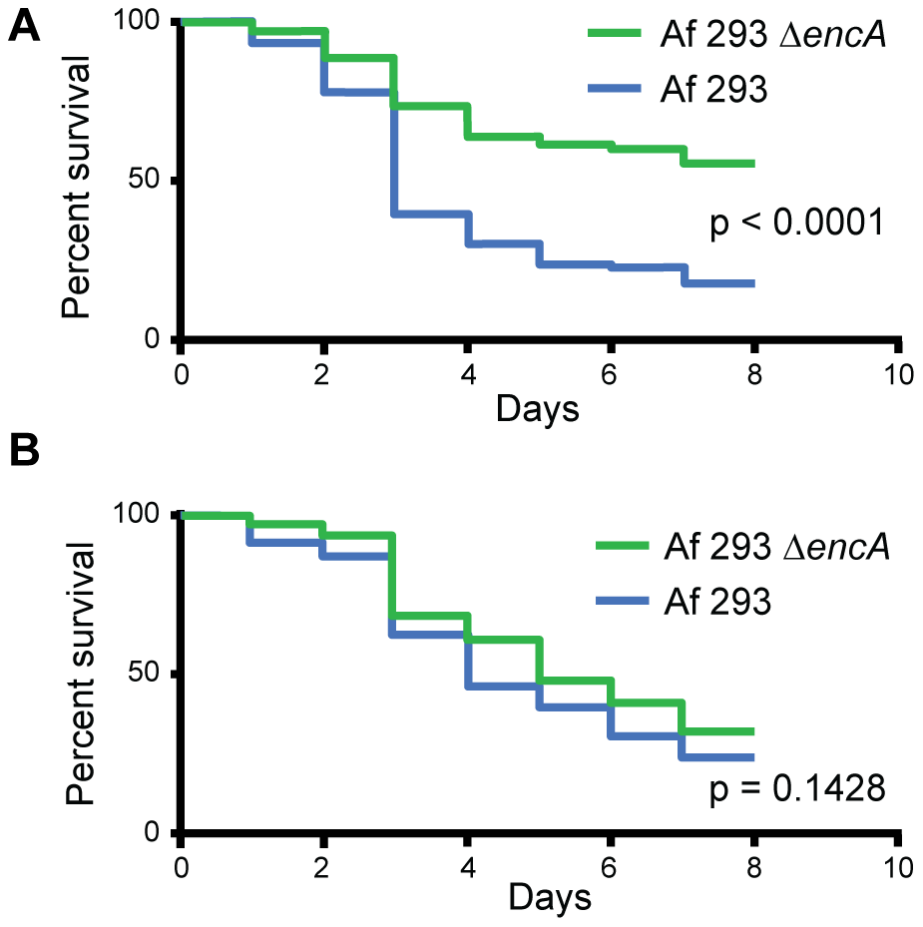
ΔencA spores exhibit attenuated virulence when harvested from endocrocin-permissive temperatures. Survival of *Drosophila* flies inoculated with WT and *ΔencA*, grown at 25°C (favorable condition for endocrocin production) (A) and 37°C (unfavorable condition for endocrocin production) (B).

Finally we assessed the pathogenicity attribute(s) of products from the endocrocin gene cluster *in vivo* by using the established Toll-deficient *Drosophila* invasive aspergillosis model [30], [31]. This model, while not possessing the immune system of mammalians, is utilized successfully for studying virulence of *A. fumigatus* and, importantly, meets the affordable and rapid methodology of this study. *Drosophila* were inoculated with *A. fumigatus* wild-type and *ΔencA* spores harvested from 25°C (endocrocin stimulating) or 37°C (endocrocin restrictive) environments. Attenuated virulence was observed when flies were inoculated with spores of *ΔencA* grown at 25 but not 37°C, consistent with the observed temperature-dependent production of endocrocin (Figure 6A,B).

## Discussion

Modern metabolomic techniques provide the ability to identify and isolate hundreds of microbial compounds in academic research settings. Downstream of these techniques, there is an increasing need for low-volume platforms that can help map the multi-kingdom interactome in a low-cost laboratory setting. We present here a novel and economical approach for identifying immunosuppressive properties of microbial compounds. The two-tiered approach offers an accessible solution for performing a screen both *in vitro*, on human primary neutrophils, and *in vivo*, on whole organism zebrafish models. Both methods described are compatible with natural product extraction methods with small yields and are scalable as they allow batch processing. The combination of assessing the response of human primary cells - a more precise model for medical studies - and using zebrafish for a more holistic *in vivo* assessment of specific innate immune responses, covers a highly relevant scope of models for predicting the effect of metabolites in humans.

While a multitude of microfluidic gradient and neutrophil migration platforms have been demonstrated to date, this is the first systematic assessment of the neutrophil migration properties of Aspergilli SMs in a microfluidic embodiment. It is worthy to note, however, that due to material interaction and a higher surface-to-volume ratio in microscale devices, it is possible for the compound tested to be sequestered by the material and not be able to diffuse to the neutrophils, leading to false negatives. Using this microscaled approach, we find that four of the compounds tested displayed migration inhibitory properties. The *in vivo* zebrafish assay complements the *in vitro* assay as it uses an equally scalable and potentially automatable approach, as specific manual treatment of the fish used for each compound is not necessary. One potential caveat is the necessity for the compound to traverse the skin barrier in order to have an effect on the neutrophils. In this aspect, the assay can be made more sensitive by pre-incubating the fish in the purified compounds prior to adding the chemoattractant. Together, these assays represent a new avenue for the discovery of immunosuppressive properties of microbial SMs. As an example, we found that one of the compounds selected from the *in vitro* screen, endocrocin, displayed significant leukocyte recruitment inhibitory properties.

Endocrocin was only recently identified as a LaeA regulated SM in *A. fumigatus* [28]. Coupling the observations that *laeA* loss yields reduced virulent strains and that several studies have shown uncharacterized components of *A. fumigatus* filtrates inhibit neutrophil chemotaxis, we suggest that endocrocin is one of these components. The fact that endocrocin did not result in zebrafish death nor increase neutrophil death under the assay conditions used (Figure S1) further supports a more specific function for this metabolite than mere cytotoxicity.

The study of endocrocin showed that it is located in the fungal spores, and may be released upon contact with a humid environment such as the host lung tissues or upon early germination of spores that managed to evade macrophage clearance. Previous studies have found that certain fungal metabolites, associated with the cell wall of the inhaled spores, have the ability to interact detrimentally with the lung epithelia [32], [33]. This study provides insight into the role of spore-borne metabolites as “protective” constituents during early lung colonization, in which the spores are pre-armed with metabolites that could provide the germinating spore with an advantage over the immune system. Spores containing these “protective” attributes that could modulate or negate early host innate confrontations – the first line of defense towards *Aspergillus* infections – may provide the fungus with leverage during this initial host-pathogen arms race. Thus, spore-borne metabolites, highly dependent on the micro-environmental conditions in which the spores originate (temperature, nutrient sources, microbial interactions, *etc*) may play an important and understudied role in the pathomechanism of fungal opportunistic diseases.

## Materials and Methods

### Purification of the SM library

Purified fungal metabolites were obtained by culturing *Aspergillus* species (*A. fumigatus* and *A. nidulans* wild-type and mutant stains) in liquid shake or solid agar conditions. For the liquid shake culture, the supernatant was collected and small molecules extracted by freeze drying and methanol extraction. For solid agar cultures, the agar was homogenized and soaked in 800 mL of 1∶1 CH_2_Cl_2_/Methanol for 24 h. After filtration, the combined extract was evaporated *in vacuo* to yield a residue, which was suspended in water (500 mL) and then partitioned with ethyl acetate (500 mL) three times. The combined ethyl acetate layer was evaporated *in vacuo* to afford a crude extract (the weight for each deletant is list below). The crude extract was applied to a Si gel column (Merck, 230 to 400 mesh, ASTM, 20×80 mm) and eluted with 250 mL CH_2_Cl_2_/Methanol mixtures of increasing polarity (fraction A, 1∶0; fraction B, 19∶1; fraction C, 9∶1; fraction D, 7∶3). Each fraction was examined by high performance liquid chromatography–photodiode array detection–mass spectrometry (HPLC–DAD–MS) and the fraction contained target natural products was applied to a gradient HPLC on a C18 reverse phase column (Phenomenex Luna 5 µm C18 [2], 250×10 mm) with a flow rate of 5.0 mL/min and measured by a UV detector at 254 nm. The gradient system was acetonitrile (solvent B) and 5% Acetonitrile/H_2_O (solvent A) both containing 0.05% Tetra-Fluoroboric Acid.

### Neutrophil preparation

The neutrophils were purified from whole blood of consenting self-reported healthy donors. A volume of 7 mL of whole blood was placed in a 15 mL conical tube and 7 mL of Polymorphoprep liquid (Axis Shield, UK) was layered above. The conical tube was centrifuged for 20 min at 1200 rpm followed by 10 min at 1700 rpm. The neutrophil layer was removed, placed in a 50 mL conical tube, and 1× PBS was added to fill the tube up to 50 mL. The conical tube was subsequently centrifuged for 10 min at 1500 rpm. The pellet was re-suspended in 9 mL of de-ionized H_2_O for 30 s after which 1 mL of 10× PBS and 40 mL of 1× PBS were added. The conical tube was centrifuged again for 10 min at 1500 rpm, and the pellet was re-suspended in 1 mL of PBS. The neutrophils were counted and the cell suspension was diluted to a final concentration of 4 million per mL.

### Microfluidic gradient-generation devices

A silicon-based mold for the microfluidic device-arrays was fabricated by creating the design on Illustrator (Adobe, USA), and printing on a high-resolution film (Imagesetter, Inc, Madison, USA). The main channels of the microfluidic device were designed to be 1 mm wide and 400 µm tall, while the gradient channel is 35 µm tall, 150 µm wide, and 1 mm long. A photo sensitive epoxy, SU-10 (Microchem, USA), was spun on a 150 mm diameter wafer (WRS, USA) to a thickness of 35 µm and backed for 10 min at 95°C. The first film defining the migration channels was placed on the wafer and exposed to 200 mJ of UV light from an Omnicure light source (EXFO, Canada), after which the wafer was post-backed at 95°C for 5 min. A second layer of epoxy, SU-100, was spun on the wafer to a thickness of 400 µm, backed for 90 min at 95°C, exposed with 1200 mJ of UV light with the second film defining the microfluidic channels, and backed for 30 min. A third layer of SU-100 was spun and exposed with the same parameters as the second layer, albeit with the third film defining the ports. The wafer was developed in SU-developer (Microchem, USA) for 3 hours on a shaker, washed with acetone, rinsed with iso-propanol, and dried using compressed air.

Microfluidic arrays were designed and fabricated using silicone polymer-based soft-lithography methods by replicating a silicon-SU8 master mold [34]. In brief, a silicone polymer, PDMS (Sylgaard 184, Dow Corning, USA), was mixed with a ratio of 1∶10 of curing agent to monomer base and placed in a vacuum for 30 min for degassing. The molding process was performed by placing, in order, on a hot plate a transparency sheet (Cheap Joe's, USA), the silicon-based mold, degassed PDMS, a second transparency sheet, a 1 mm thick layer of silicon foam (Mc Master, USA), a rectangle of glass, and a 5 kg weight. The hot plate was heated to 80°C for 3 hours and cooled down completely prior to PDMS removal. The PDMS layer was peeled off the silicon-based mold, bonded non-covalently in a polystyrene Omnitray dish (NUNC, USA). The Omnitray was filled with 20 mL of PBS and placed in a vacuum chamber for 15 min in order to fill the microdevices. The superfluous PBS was removed, 4 µL of mHBSS (1× PBS containing 0.1% HSA and 0.2 mM of HEPES buffer) were placed on the output well and 4 µL were placed on each input well in order to replace the fluid contained in each device.

Purified dried compounds were resuspended in DMSO to 1 mM and subsequently diluted 1∶100 in mHBSS containing 100 nM of fMLP – a known chemoattractant. 4 µL of neutrophil suspension at 4.10^6^ cells per mL was inserted into the sink channel of the device, followed by 3 µL of compound in the source channel, and the Omnitrays were placed in a CO_2_ incubator. After 45 min, the Omnitrays were removed, placed on a Nikon Eclipse microscope, and phase-contrast images of the migration channels were taken at 10× magnification. Image analysis was performed using the software package Je'Xperiment developed in-house (source code available on sourceforge.net or upon request) allowing the identification and quantification of the neutrophils invading the migration channel on a batch processing scale. A Wilcoxon rank-sum test was performed on sets of 5 data points to evaluate the statistical significance of the effect of each compound. Those with a p-value of less than 0.05 were considered statistically significant.

### Zebrafish purified SM experiment

Zebrafish were maintained according to the protocols approved by the University of Wisconsin-Madison Research Animal Resources Center. Zebrafish larvae at 3 days post fertilization were placed in the wells of a 24 well-plate (20 per well). The larvae were pre-incubated in 500 µL of E3 (egg water) containing 10 µM of the purified fungal compound in DMSO. After 1 hour, LTB_4_ was added to each well at a final concentration of 30 nM. The larvae were fixed after 30 min and stained using Sudan Black [26]. Numbers of neutrophils recruited to the ventral fin of each fish were counted manually and representative images were taken with a phase contrast upright microscope. Statistical significance was estimated using a Kruskal-Wallis test followed by Dunn's Multiple Comparison test in the software GraphPad Prism (USA). The animal handling protocols were performed according to the Guide of the Care and Use of Animals of the National Institutes of Health.

### Culture conditions, endocrocin extraction, TLC analysis

*Aspergillus fumigatus* strains used in this study are listed in Table S1. Strains were maintained as glycerol stocks and activated on glucose minimal media (GMM). Conidia were harvested in 0.01% Tween 80 and enumerated using a hemocytometer. Strain TFYL7.1 was constructed by targeted gene deletion of *encA* using a deletion cassette made via double-joint fusion PCR described in [29]) into strain AF293.1. Internal primers to *encA* was used to confirm the absence of its open reading frame and single integration of the deletion cassette was confirmed via Southern analysis using two different restriction digest profiles as described in [29]) (data not shown).

For temperature-dependent characterization of endocrocin production, *A. fumigatus* WT and *ΔencA* strains were point-inoculated at 1×10^4^ conidia/inoculum onto solid GMM and incubated at temperatures ranging from 25–42°C without light selection. A 1.2 cm diameter core was removed from the middle of the fungal culture and homogenized in 2 mL of 0.01% Tween 80. The homogenized mixture was extracted with equal volumes of ethyl acetate and routine vortexing at room temperature over the course of 30 min. The mixture was centrifuged for 5 min at 3,500 rpm and 1 mL of the ethyl acetate layer was removed and allowed to evaporate at room temperature to yield a dried crude extract. For TLC analysis, the crude extract was reconstituted in 50–100 µL of ethyl acetate and 5–10 µL was spotted onto a 250 µM analytical silica plate (Whatman, Cat 4410-222) and subjected to a toluene: ethyl acetate: formic acid (5∶4∶0.8) resolving phase. Plates were visualized at 366 nm using a FOTO/Analyst® Investigator gel imaging system (Fotodyne Inc).

For tissue specific extraction of endocrocin, a suspension of 1×10^6^ conidia in molten GMM top agar (0.75% w/v agar) was overlaid over solid GMM bottom agar (1.5% w/v agar). SM from different developmental parts of the fungus were obtained as follows: conidia was obtained by gently tapping the fungal culture with the petri dish lid side down as described previously [35], The remaining conidia and conidiophores were removed by gently scraping the surface of the fungal culture with 0.01% Tween 80 followed by multiple washes to remove residual conidia/conidiophores. The mycelia (free of aerial conidiophores/conidia) were obtained by peeling off the top agar from the culture plate. The bottom agar that is now free of mycelia (as inspected under the microscope) was extracted. A diagram that depicts the various sampling parts of the culture plate can be found in Figure 3B. Extraction process and TLC analyses were performed as described above.

### Automated neutrophil counting

We developed an image-processing algorithm that is able to identify un-labeled neutrophils in a phase-contrast image and quantify their number of migration distance (Figure1F). Used in conjuncture with a software platform developed in-house, called Je'Xperiment (Source code available on sourceforge.net or on request), which allows batch processing and data mining, we show a first step towards creating a high-throughput solution for quantifying the effect of fungal SMs on the inhibition of leukocyte migration.

### Drosophila survival experiments

Flies were generated by crossing flies carrying a thermosensitive allele of Toll (Tl r632) with flies carrying a null allele of Toll (Tl I-RXA) [36]. Two- to four day old adult female Toll-deficient flies were used in all of the experiments. Twenty flies were infected with each *A. fumigatus* strain used in this study. *A. fumigatus* isolates (*ΔencA* and wild-type) were grown on yeast extract agar glucose (YAG) either at 37°C or 25°C. Conidia were collected in sterile 0.9% saline from 2 days old cultures. The conidial concentration suspension was determined by using a hemacytometer and adjusted to 1×10^8^ per mL. The dorsal side of the thorax of 20 CO_2_ anesthetized flies was punctured with a thin (10 µm) sterile needle that had been dipped in a concentrated solution of *A. fumigatus* conidia (10^7^ spores/mL). As a negative control group, Toll-deficient flies were punctured with a 10 µm sterile needle and monitored daily for survival. Flies that died within 3 h of the injection were considered to have died as a result of the procedure and were not included in the survival rate analysis. The flies were housed in a 29°C incubator to maximize expression of the Tl r632 phenotype [31]. The Toll-deficient flies were transferred into fresh vials every 3 days. Fly survival was assessed daily over 10 days. Each experiment was repeated 3 times on different days and at the same time of the day to eliminate variability due to circadian rhythm.

### Ethics statement

Neutrophils were obtained from whole blood of self-reportedly healthy donors, from which we obtained informed and written consent at the time of the blood draw with approval of the University of Wisconsin-Madison Center for Health Sciences Human Subjects committee.

## Supporting Information

Figure S1 **Live/Dead assay on neutrophils treated with Endocrocin.** A. Fluorescent microscopy image of a live/dead stain of neutrophils treated with Endocrocin and a negative control. B. Quantification of the ratio of live and dead neutrophils in the fluorescent images.
(TIF)

Movie S1 **Timelapse microscopy of neutrophil recruitment into the tail fin of a zebrafish drowsed in the chemoattractant LTB_4_.**
(MOV)

Movie S2 **Timelapse microscopy of neutrophil recruitment into the tail fin of a zebrafish drowsed in the chemoattractant LTB_4_ and a known neutrophil inhibitor, LY294002.**
(MOV)

Table S1 ***A. fumigatus*** **strains used in this study.**
(DOCX)
