# Supplementary figures and images for: Low-Volume Toolbox for the Discovery of Immunosuppressive Fungal Secondary Metabolites
Source: PLoS Pathog. 2013 Apr 11;9(4):e1003289. doi: 10.1371/journal.ppat.1003289 (PMC3623715; doi:10.1371/journal.ppat.1003289)

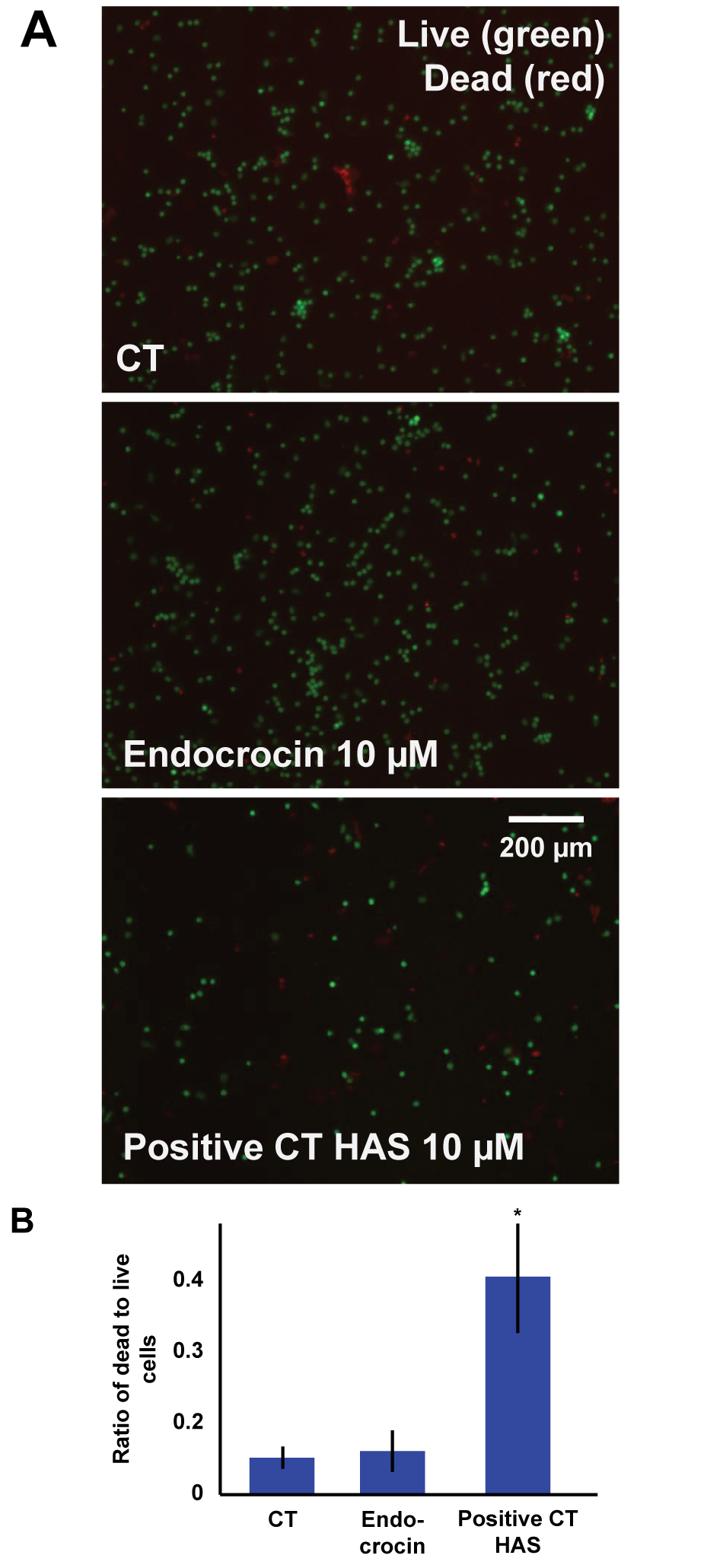

Supplement: Figure S1 — Live/Dead assay on neutrophils treated with Endocrocin. A. Fluorescent microscopy image of a live/dead stain of neutrophils treated with Endocrocin and a negative control. B. Quantification of the ratio of live and dead neutrophils in the fluorescent images. (TIF) [file ppat.1003289.s001.tif]
