# Supplementary material for: Low-Volume Toolbox for the Discovery of Immunosuppressive Fungal Secondary Metabolites
Source: PLoS Pathog. 2013 Apr 11;9(4):e1003289. doi: 10.1371/journal.ppat.1003289 (PMC3623715; doi:10.1371/journal.ppat.1003289)
Supplement: Table S1 — A. fumigatus strains used in this study. (DOCX) [file ppat.1003289.s004.docx]

## .

| **Strain** | **Genotype** | **Source** |
| --- | --- | --- |
| CEA17 KU80 pyrG+ | *ΔnkuB::A. fumigatus pyrG* | [34] |
| TFYL6.3 | *ΔnkuB::A. fumigatus pyrG; ΔencA::A.parasiticus pyrG* | [27] |
| TFYL1.51 | *ΔnkuB::A. fumigatus pyrG; A.parasiticus pyrG::gpdA(p)::encA* | [27] |
| AF293 | *Wild type* | [36] |
| AF293.1 | *pyrG1* | [36] |
| TFYL7.1 | *ΔencA::A.parasiticus pyrG; pyrG1* | This study |

## 
